# Effects of Soil Characteristics, Allelopathy and Frugivory on Establishment of the Invasive Plant *Carpobrotus edulis* and a Co-Occuring Native, *Malcolmia littorea*

**DOI:** 10.1371/journal.pone.0053166

**Published:** 2012-12-28

**Authors:** Ana Novoa, Luís González, Lenka Moravcová, Petr Pyšek

**Affiliations:** 1 Departamento de Bioloxía Vexetal e Ciencia do Solo, Universidade de Vigo, Vigo, Spain; 2 Department of Invasion Ecology, Academy of Sciences of the Czech Republic, Průhonice, Czech Republic; 3 Department of Ecology, Charles University in Prague, Prague, Czech Republic; Institut Mediterrani d'Estudis Avançats (CSIC/UIB), Spain

## Abstract

**Background:**

The species *Carpobrotus edulis*, native to South Africa, is one of the major plant invaders of Mediterranean coastal ecosystems around the world. Invasion by *C. edulis* exerts a great impact on coastal habitats. The low number of native species in invaded communities points to the possible existence of mechanisms suppressing their germination. In this study we assessed whether soil factors, endozoochory, competition and allelopathic effects of the invader affect its own early establishment and that of the native species *Malcolmia littorea*. We used laboratory solutions representing different chemical composition and moisture of the soil, herbivore feeding assays to simulate seed scarification and rainwater solutions to account for the effect of differently aged *C. edulis* litter.

**Principal Findings:**

We show that unlike that of the native species, germination and early growth of *C. edulis* was not constrained by low moisture. The establishment of *C. edulis*, in terms of germination and early growth, was increased by scarification of seeds following passage through the European rabbit intestines; the rabbits therefore may have potential implications for plant establishment. There was no competition between *C. edulis* and *M. littorea*. The litter of the invasive *C. edulis,* which remains on the soil surface for several years, releases allelopathic substances that suppress the native plant germination process and early root growth.

**Conclusions:**

The invasive species exhibits features that likely make it a better colonizer of sand dunes than the co-occurring native species. Allelopathic effects, ability to establish in drier microsites and efficient scarification by rabbits are among the mechanisms allowing *C. edulis* to invade. The results help to explain the failure of removal projects that have been carried out in order to restore dunes invaded by *C. edulis*, and the long-lasting effects of C. edulis litter need to be taken into account in future restoration projects.

## Introduction

Coastal dunes are very dynamic and highly fragile ecosystems that are constantly evolving and changing, which imposes marked selection pressures resulting in a high degree of specialization of coastal dune plants [1]. Therefore, these ecosystems have a high cultural and ecological value, and support many threatened and endemic species [2]. Currently, coastal dune ecosystems are damaged as a result of human influences such as construction and leisure activities, as well as climate change, resulting in severe habitat change, over-exploitation and nitrogen pollution. However, a major part of this threat can be attributed to invasions by alien plants, which are considered to be one of the greatest threats to the diversity, structure and functioning of natural ecosystems around the world [3], [4], [5], [6], [7], [8], including the Mediterranean region [9], [10]. Their impact on costal dune ecosystems has been evaluated as high and still increasing according to the Millennium Ecosystem Assessment [7]. Thus, to conserve the coastal Mediterranean ecosystems it is necessary to mitigate the impacts of plant invasions.

Alien plants must successfully pass through several stages to become invasive [11], that include introduction to the new range, where abiotic conditions promote colonization of local habitats [12], [13], and successful establishment and dispersal (e.g. [14], [15], [16], [17], [18], [19], [20], [21]). Therefore, to mitigate the impacts of plant invasions on Mediterranean coastal ecosystems, one needs to understand the mechanisms that facilitate the success of the most serious invasive species, including the influences of native soil factors, dispersal mechanisms, competition relationships with native species, or potential allelopathy.

One of the major plant invaders of Mediterranean coastal dune ecosystems is *Carpobrotus edulis* (L.) N. E. Br. (Aizoaceae), a perennial clonal and succulent plant native to South Africa [22], [23]. It is among the most intensively studied invasive plants globally [24], and those the impacts of which are often addressed [25]. When *C. edulis* invades coastal habitats [26], it modifies certain soil parameters [27], [28], [29], [30], which can have a great impact on community composition, diversity and succession [31], [32]. Some of these changes, such as in moisture content, pH, and salinity, influence the germination and early growth of native plants (Novoa and González, unpublished) as well as its own performance [33], and persist following the removal of the invader [34], [35].Thus, an improved understanding of the interactions between those factors is crucial for a better mitigation of the impacts caused by *C. edulis* in the Mediterranean ecosystems.

Once established, *C. edulis* produces a fleshly indehiscent fruit in early spring, which remains on the plant until autumn when it is eaten by a variety of native mammals [36]. Its distribution is to a large extent determined by humans acting as dispersal agent, using it as an ornamental plant or for dunes stabilization [23]. But nowadays, the endozoochor dispersal by unspecialized consumers may also help to explain its success as an invader [37], [38].

Moreover, allelopathic interactions between alien and native species are one possible strategy for the success of plant invaders [39], [40], [41]. The interaction between environmental soil factors and allelopathic effects of invasive plants has been repeatedly documented ([42], see [43] for review) but never studied in *C. edulis.* Thus, in order to properly restore previously invaded ecosystem after the removal of *C. edulis*, it is important to explore the role of the possible allelopathic effect of the litter that remains on the soil after the invasive species has been removed.

Since the interactions between invasive and native species at the germination stage of population development can principally affect the invasion success of *C. edulis*, and the recolonization of previously invaded ecosystems, we investigated several factors hypothesized to play a role in this species' germination rate and early root growth. We also compared how these same factors affect a co-occurring annual native species, *Malcolmia littorea* (L.) R. Br., which is an endangered species in Italy and some regions of Spain [44], [45]. We generated a series of hypotheses based on the following premises: (i) *C. edulis* changes the quality of invaded microsites, influencing the establishment of native plants (Novoa and González, unpublished). (ii) The presence of *Oryctolagus cuniculus* L. (European rabbit) [39] could contribute to *C. edulis* success [25] as an invader in the studied area; this effect of rabbit was reported to interact with that of rats on offshore islands in southeast France, facilitating invasions by invasional meltdown processes [46]. (iii) There could be allelopathic and competitive interactions between *C. edulis* litter and native dune species [42]. (iv) Efforts to eradicate *C. edulis* and restore dunes, have failed (personal observation). Three years after the campaign to eliminate *C. edulis* the restored areas are reinvaded by *C. edulis* growing from seeds. Based on this information, we predicted that (i) there are potentially synergistic effects between soil pH, soil moisture and salinity in early competition between the native and the exotic species, (ii) European rabbits contribute to *C. edulis* spread, (iii) there are competitive relationships established between *C. edulis* and *M. littorea* seeds, and (iv) *C. edulis* litter exerts allelopathic effects on native plants and possibly on its own seeds. Our research objective was to gain deeper insights into how soil characteristics, seed dispersal, competition and allelopathy affect the colonization of coastal habitats by one of the most serious invasive species in Mediterranean ecosystems.

## Materials and Methods

### Study species

In many temperate coastal habitats, two species of the genus Carpobrotus co-occur: *C. edulis* and *C. acinaciformis* [47], differing in the color of the filaments of stamens [48]. Only the invasive species *C. edulis* (with yellow filaments) was found in our study area. *Carpobrotus edulis* is a perennial species native to South Africa [49], which finds climatically and ecologically suitable habitats in many coastal regions of the world [50]. It was originally introduced to Europe, California and Australia to stabilize coastal sand dunes and as an ornamental plant in early 20^th^ century [51] and has become invasive in natural habitats in western and southern Europe, the Azores, south Atlantic islands, western USA, Australia, and naturalized in Northern Africa, the Canary Islands, Madeira, southeastern USA and New Zealand [52]. Due to extensive clonal growth, it forms dense mats, displaces native vegetation and its invasion potential may be increased by hybridization in some regions [38], [53], [54], [55]. The species reproduces and spreads by seed but it is not the only mechanism responsible for its dispersal since it also spreads vegetatively by stem and leaf fragments especially where there is natural gravity gradient or where dunes are disturbed by natural temporary water streams.

*Malcolmia littorea* is an annual species native to South Europe, growing along coastal sand dunes [56]. It is distributed in France, Spain, Italy, and Portugal [22], growing in the same habitats as *C. edulis* [50]. It is an endangered species in some regions of Spain (not in the study area) [57], as well as in Italy where it was recently suggested to enhance its threat status [45] Whe chose this species because is one of the most common native plants in the area studied.

### Seed collection and preparation for the germination experiment

Seeds of the native species *Malcolmia littorea* and invasive *Carpobrotus edulis* were collected between 10^th^ September and 10^th^ October 2011 from at least 15 plants from 20 different populations of each species located along 20 km in Pontevedra Coast, Spain (between 42°29′56.17″N 8°52′16.22″O and 42°20′16.22″N 8°49′41.17″O). The seeds were separated from the rest of the fruit and its accessory dispersion parts and stored in the dark at 4°C until assay. We did not collect seed in any privately-owned or protected area and no specific permits were required for the field studies performed.

Two scarification treatments of the seeds of *C. edulis* were defined as follows: (i) non-scarified seeds and (ii) seed scarified by endozoochry. A subset of the *Carpobrotus* seeds was mixed with food for European rabbits (*O. cuniculus*). After passing through the digestive tract of rabbits, the seeds were removed from the excrement without contact with the animals. Rabbits did not suffer any damage since the method followed was simply feed them and collect the excrements. The procedure followed in the experimental design did not interfere with the assumptions showed by the European Union Council (86/609/EU) and the Spanish government (RD/1200/2005) for animal care and use. The animal housing are agree with the Spanish government (RD/348/2000) by which incorporates the legal Directive 2010/63/EU on the protection of animals on farms livestock and modified by RD/441/2001.

Seeds were surface-sterilized for 5 min in 0.1% sodium hypochlorite, rinsed 3 times in distilled water and dried at room temperature prior to the experiment to avoid fungal attack.

### Irrigation solutions

Natural solutions (from rainwater) and laboratory solutions with distilled water were obtained. To collect rainwater we established plots (42°28′37.6″N, 08°51′29.8″W) in sites without historical episodes of *Carpobrotus* invasion, covered with native vegetation (N), those from which *Carpobrotus* was removed at the beginning of the experiment, prior to rainwater collection (C0), from which *Carpobrotus* was removed 1.5 years before the beginning of the experiment (C1) and from which it was removed 3 years before the beginning of the experiment (C3). These sites were not privately-owned or protected in any way. At the time of sampling, there was still almost no vegetation in sites C0, C1 and C3, except for some *C. edulis* seedlings starting to establish there. Three traps per type of site were buried into the soil to collect water. Surface substrate (litter and soil) was removed carefully from a quadrat of 45×35×2 cm and kept, as was the sand below up to 10 cm in depth. A plastic tray (40×25×6 cm) protected with nylon net (mesh 1×1 mm) was placed in the hole, and covered with the surface substrate. Rainwater that passed through the surface substrate was accumulated into the tray, collected and kept refrigerated. This provided us with a gradient of the rainwater treatments assumed to represent the strength of the previous effect of *Carpobrotus* on soil from which the rainwater was collected. Three replicates per plot were sampled. pH and conductivity of rainwater solutions were determined [1], [58].

Laboratory solutions of different pH levels and salinities were prepared to mimic the values found in the study area in native (pH 8.5 and 0.02 gNaCl/L) and invaded (pH 7.5 and 0.04 gNaCl/L) sites (Novoa et al. unpublished).We used 12 different irrigation treatments. Four from collected rainwater: (i) rainwater from N (1.5 mL/week), (ii) rainwater from C0 (1.5 mL/week), (iii) rainwater from C1 (1.5 mL/week), (iv) rainwater from C3 (1.5 mL/week). Eight solutions were prepared in the laboratory: (v) distilled water pH 7.5, 0.02 g NaCl/L (1 mL/week), (vi) distilled water 7.5, 0.04 (1 mL/week), (vii) distilled water 8.5, 0.02 (1 mL/week), (viii) distilled water 8.5, 0.04 (1 mL/week), (ix) distilled water 7.5, 0.02 (2 mL/week), (x) distilled water 7.5, 0.04 (2 mL/week), (xi) distilled water 8.5, 0.02 (2 mL/week), (xii) distilled water 8.5, 0.04 (2 mL/week). Treatments (v) to (viii) represented low moisture conditions, (ix) to (xii) high moisture conditions.

### Germination experiment

Seeds (from the mixed sample collected in the field) of *M. littorea* and *Carpobrotus* (scarified or not scarified by European rabbits) were placed on Petri dishes (diameter 5 cm) lined with filter paper. The seed competition treatment consisted of 10 seeds in a Petri dish representing controls (*Malcolmia*; scarified *Carpobrotus*; unscarified *Carpobrotus*) and mixtures of 5 seeds each of *Malcolmia*+scarified *Carpobrotus*, or *Malcolmia*+unscarified *Carpobrotus*). Five replicates of each treatment (controls and mixtures at each irrigation level) were placed in germination chambers with periods of 12 hours of light and 25°C/15°C (temperatures and light regimes similar to those in the field). In total, 300 Petri dishes were used: [4 rainwater treatments×(2 competition+3 controls)×5 replicates]+[8 irrigation treatments×(2 competition+3 controls)×5 replicates]. The germination experiments were performed at the Institute of Botany AS CR in Průhonice.

The number of germinated seeds was recorded every second day over three weeks. At the end of the experiment, root length of five random seedlings per Petri dish was measured using caliper.

### Germination indices

Total germination rate (Gt) and the cumulative rate of germination (As) were calculated using germination data. These indices are representative of the germination patterns [59]. The total germination (Gt) provides an overview of the germination process. It detects possible stimulatory or inhibitory effects on germination, and reports the germination capacity of each species in each situation [60]. Gt = (Nt×100/N), where Nt is the total number of seeds germinated at the last measurement time and N is the number of seeds used in the bioassay. The Speed of Cumulative Germination index (AS) indicates the effect of treatment on the cumulative speed during each of the times [61], [62]. AS = (n_1_/1+n_2_/2+n_3_/3+…+n_n_/n), where n_1_,n_2_, n_3_… n_n_ are the cumulative number of germinated seeds at time 1, 2…n throughout the assay.

### Statistical analysis

Data were analysed with the statistical program IBM - SPSS Statistics 19 (SPSS, Inc., Chicago, IL). The first exploratory analysis of the data was performed using box plots to detect and remove outliers. After the outliers were removed, we applied the Kolmogorov-Smirnov test to check the normality of the data, and the Levene test for homogeneity of variances to test their homoscedasticity. The data met conditions of normality and homoscedasticity and thus were analyzed using a simple factorial analysis of variance (ANOVA) and Tukey test [63] for multiple comparisons.

**Figure 1:**
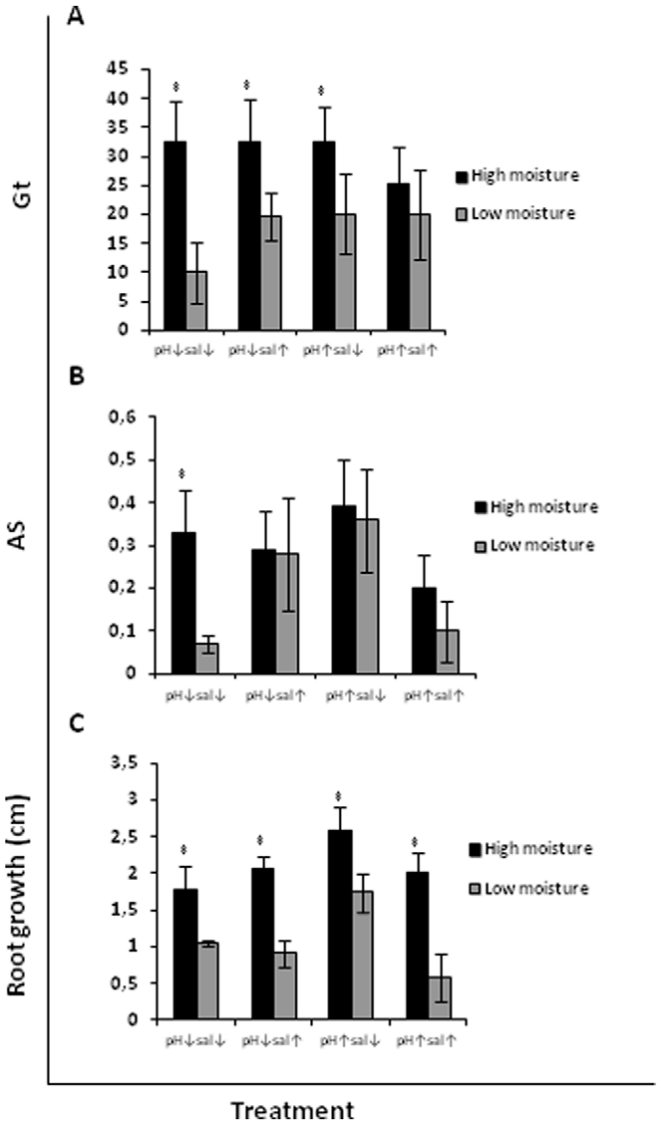
Total germination rate Gt (A), cumulative germination AS (B) and root growth (C) of the native species *Malcolmia littorea* as affected by moisture level.

**Figure 2:**
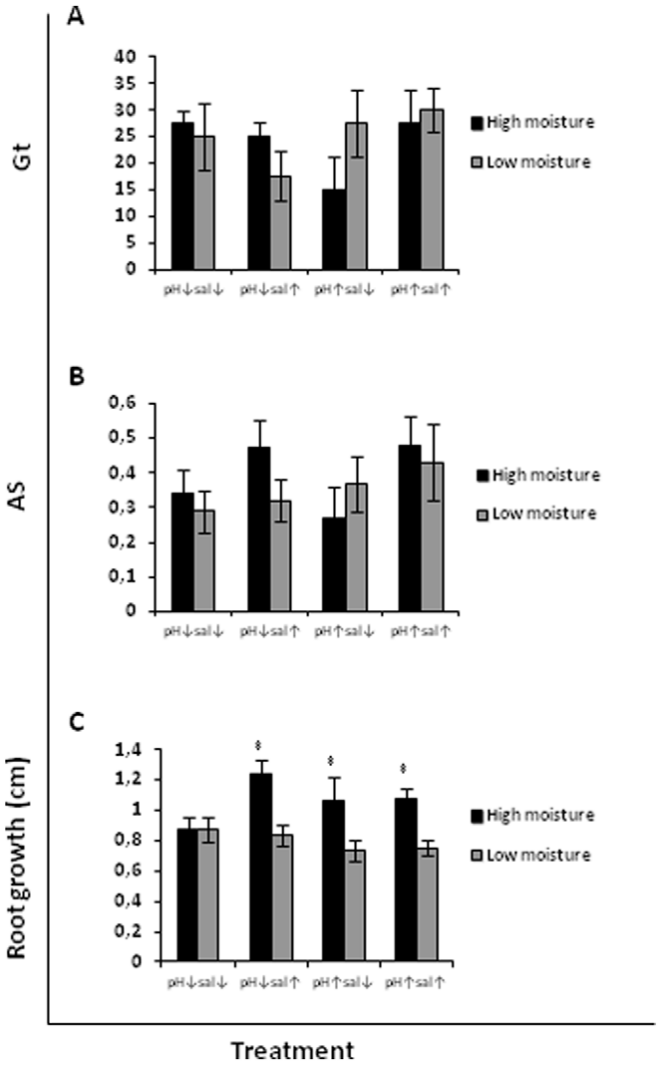
Total germination rate Gt (A), cumulative germination AS (B) and root growth (C) of the invasive species *Carpobrotus edulis* without scarification as affected by moisture level.

**Figure 3:**
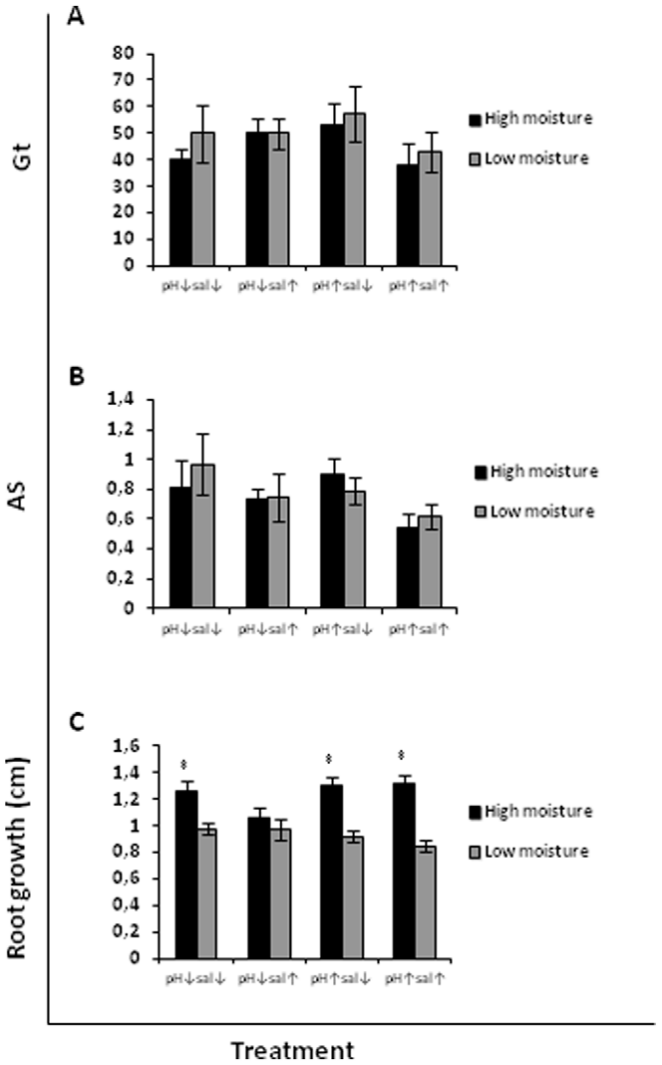
Total germination rate Gt (A), cumulative germination AS (B) and root growth (C) of the invasive species *Carpobrotus edulis* scarified as affected by moisture level.

Prior to the main analyses, a preliminary test was done with a three-way ANOVA, using moisture, pH and salinity (low and high), and scarification (*C. edulis* seeds eaten and uneaten by rabbits) as factors (Figures 1 and 2; Tables 1 and 2). We also conducted a two-way ANOVA using rainwater treatments (N, C0, C1 and C3) and scarification as factors (Figure 3; Table 3). But, there were no significant interactions we do not show the results of the preliminary tests here.

**Table 1:** Total germination rate (Gt), cumulative germination (AS) and root growth of the native species *M. littorea* as affected by salinity and pH.

	Salinity							
	pH↓moist↓		pH↓ moist ↑		pH↑ moist ↓		pH↑ moist ↑	
	High salinity	Low salinity	High salinity	Low salinity	High salinity	Low salinity	High salinity	Low salinity
Gt	19.7	10.0	32.5	32.5	20.1	20.0	25.4	32.5
	(4.0)	(5.2)	(7.4)	(7.1)	(7.8)	(6.9)	(6.4)	(6.2)
AS	0.28	0.12	0.28	0.33	0.13	0.32	0.20	0.39
	(0.13)	(0.09)	(0.09)	(0.10)	(0.07)	(0.12)	(0.08)	(0.11)
Root	0.91	1.05	2.07	1.81	0.89	1.55	2.00	2.36
	(0.19)	(0.05)	(0.15)	(0.33)	(0.32)	(0.26)	(0.28)	(0.32)

	pH							
	sal↓moist↓		sal↓moist↑		sal↑moist↓		sal↑moist↑	
	High pH	Low pH	High pH	Low pH	High pH	Low pH	High pH	Low pH
Gt	20.0	10.0	32.5	32.5	20.1	19.7	25.4	32.5
	(6.9)	(5.2)	(6.2)	(7.1)	(7.8)	(4.0)	(6.4)	(7.4)
As	0.32	0.12	0.39	0.33	0.13	0.28	0.20	0.28
	(0.12)	(0.09)	(0.11)	(0.10)	(0.07)	(0.13)	(0.08)	(0.09)
Root	1.55	1.05	2.36	1.81	0.89	0.91	2.00	2.07
	(0.26)	(0.05)	(0.32)	(0.33)	(0.32)	(0.19)	(0.28)	(0.15)

There are no significant differences at 5% level between the seeds treated with high (0.04 g NaCl/L) and low (0.02 g NaCl/L) salinity or high (7) and low (8) pH. Watered conditions: pH↓moist↓: pH 7, 1 mL/week; pH↓moist↑: pH 7, 3 mL/week; pH↑moist↓: pH 8, 1 mL/week; pH↑moist↑: pH 8, 2 mL/week; sal↓moist↓: 0.02 g NaCl/L, 1 mL/week; sal↓moist↑: 0.02 g NaCl/L, 3 mL/week; sal↑moist↓: 0.04 g NaCl/L, 1 mL/week; sal↑moist↑: 0.04 g NaCl/L, 2 mL/week. Sal = salinity, moist = moisture.

**Table 2:** Total germination rate (Gt), cumulative germination (AS) and root growth of the invasive species *C. edulis* without scarification as affected by salinity and pH.

	Salinity							
	pH↓moist↓		pH↓ moist ↑		pH↑ moist ↓		pH↑ moist ↑	
	High salinity	Low salinity	High salinity	Low salinity	High salinity	Low salinity	High salinity	Low salinity
Gt	19.5	25.1	25.2	27.5	28.1	27.5	27.5	15.1
	(4.7)	(6.4)	(2.8)	(2.5)	(4.0)	(6.2)	(6.3)	(6.3)
As	0.27	0.29	0.47	0.34	0.43	0.37	0.47	0.31
	(0.06)	(0.06)	(0.08)	(0.07)	(0.11)	(0.08)	(0.08)	(0.09)
Root	0.84	0.87	1.14	0.88	0.74	0.73	1.08	1.06
	(0.07)	(0.08)	(0.09)	(0.07)	(0.05)	(0.07)	(0.06)	(0.16)

	pH							
	sal↓moist↓		sal↓moist↑		sal↑moist↓		sal↑moist↑	
	High pH	Low pH	High pH	Low pH	High pH	Low pH	High pH	Low pH
Gt	27.5	25.1	15.1	27.5	28.1	19.5	27.5	25.2
	(6.2)	(6.4)	(6.3)	(2.5)	(4.0)	(4.7)	(6.3)	(2.8)
As	0.37	0.29	0.31	0.34	0.43	0.27	0.47	0.47
	(0.08)	(0.06)	(0.09)	(0.07)	(0.11)	(0.06)	(0.08)	(0.08)
Root	0.73	0.87	1.06	0.88	0.74	0.84	1.08	1.14
	(0.07)	(0.08)	(0.16)	(0.07)	(0.05)	(0.07)	(0.06)	(0.09)

There are no significant differences at 5% level between the seeds treated with high (0.04 g NaCl/L) and low (0.02 g NaCl/L) salinity or high (7) and low (8) pH. Watered conditions: pH↓moist↓: pH 7, 1 mL/week; pH↓moist↑: pH 7, 3 mL/week; pH↑moist↓: pH 8, 1 mL/week; pH↑moist↑: pH 8, 2 mL/week; sal↓moist↓: 0.02 g NaCl/L, 1 mL/week; sal↓moist↑: 0.02 g NaCl/L, 3 mL/week; sal↑moist↓: 0.04 g NaCl/L, 1 mL/week; sal↑moist↑: 0.04 g NaCl/L, 2 mL/week. Sal = salinity, moist = moisture.

**Table 3:** Total germination rate (Gt), cumulative germination (AS) and root growth of the invasive species *C. edulis* scarified as affected by salinity and pH.

	Salinity							
	pH↓moist↓		pH↓ moist ↑		pH↑ moist ↓		pH↑ moist ↑	
	High salinity	Low salinity	High salinity	Low salinity	High salinity	Low salinity	High salinity	Low salinity
Gt	50.0	50.0	50.0	40.0	43.0	57.5	38.0	53.0
	(5.7)	(10.8)	(5.7)	(4.3)	(7.5)	(10.3)	(8.5)	(8.5)
As	0.74	0.97	0.73	0.81	0.62	0.79	0.64	0.82
	(0.16)	(0.20)	(0.08)	(0.17)	(0.08)	(0.09)	(0.10)	(0.11)
Root	0.97	0.98	1.06	1.26	0.85	0.92	1.32	1.30
	(0.08)	(0.04)	(0.08)	(0.12)	(0.04)	(0.04)	(0.06)	(0.07)

	pH							
	sal↓moist↓		sal↓moist↑		sal↑moist↓		sal↑moist↑	
	High pH	Low pH	High pH	Low pH	High pH	Low pH	High pH	Low pH
Gt	57.5	50.0	53.0	40.0	43.0	50.0	38.0	50.0
	(10.3)	(10.8)	(8.5)	(4.5)	(7.5)	(5.7)	(8.5)	(5.7)
As	0.79	0.97	0.82	0.81	0.62	0.74	0.64	0.73
	(0.09)	(0.20)	(0.11)	(0.17)	(0.08)	(0.16)	(0.10)	(0.08)
Root	0.92	0.98	1.30	1.26	0.85	0.97	1.22	1.16
	(0.04)	(0.04)	(0.07)	(0.12)	(0.04)	(0.08)	(0.06)	(0.08)

There are no significant differences at 5% level between the seeds treated with high (0.04 g NaCl/L) and low (0.02 g NaCl/L) salinity or high (7) and low (8) pH. Watered conditions: pH↓moist↓: pH 7, 1 mL/week; pH↓moist↑: pH 7, 3 mL/week; pH↑moist↓: pH 8, 1 mL/week; pH↑moist↑: pH 8, 2 mL/week; sal↓moist↓: 0.02 g NaCl/L, 1 mL/week; sal↓moist↑: 0.02 g NaCl/L, 3 mL/week; sal↑moist↓: 0.04 g NaCl/L, 1 mL/week; sal↑moist↑: 0.04 g NaCl/L, 2 mL/week. Sal = salinity, moist = moisture.

## Results

### Effects of moisture, pH and salinity

*Malcolmia littorea* germinated better at high moisture, as indicated by higher values of both germination metrics used (Fig. 1 A, B). The seed emergence was increased under high moisture at all combinations of pH and salinity; Gt values were up to 4 times greater at high than low moisture. However, the seed emergence of *C. edulis* was not influenced by moisture (Fig. 2 A, B and Fig. 3 A, B). Neither salinity nor pH had an effect on the germination of the native species *M. littorea* and the invasive species *C. edulis* (Tables 1, 2 and 3).

The root growth of *M. littorea* and *C. edulis* (from both scarified and non-scarified seed) was increased at high moisture (Fig. 1C, 2C and 3C, respectively), but for neither species was it significantly affected by pH or salinity (Tables 1, 2 and 3 respectively).

### Scarification

The germination and root growth of *C. edulis* were significantly greater following scarification by passage through the rabbits' intestines under most of the treatments (Table 4 and 5).

**Table 4:** Results of one-way ANOVA testing the effects of scarification on total germination Gt, cumulative germination AS and root growth of *Carpobrotus edulis* in pure cultures.

	High moisture								Low moisture							
	pH↓sal↓		pH↓sal↑		pH↑sal↓		pH↑sal↑		pH↓sal↓		pH↓sal↑		pH↑sal↓		pH↑sal↑	
	C	R	C	R	C	R	C	R	C	R	C	R	C	R	C	R
Gt	27.5*	40.0	25.2*	50.0	15.1*	53.0	27.5	38.0	25.1*	50	19.5*	50	27.5*	57.5	28.1	43.0
	(2.5)	(4.08)	(2.8)	(5.7)	(6.3)	(8.5)	(6.3)	(8.5)	(6.4)	(10.8)	(4.7)	(5.7)	(6.2)	(10.3)	(4.0)	(7.5)
As	0.34*	0.81	0.47*	0.73	0.31*	0.9	0.47*	0.54	0.29*	0.97	0.27*	0.74	0.37*	0.79	0.43	0.62
	(0.07)	(0.17)	(0.08)	(0.08)	(0.09)	(0.11)	(0.08)	(0.10)	(0.06)	(0.2)	(0.06)	(0.16)	(0.08)	(0.09)	(0.11)	(0.08)
Root	0.88*	1.26	1.14	1.06	1.06	1.30	1.08*	1.32	0.87	0.98	0.84	0.97	0.73*	0.92	0.74	0.85
	(0.07)	(0.08)	(0.09)	(0.08)	(0.16)	(0.07)	(0.06)	(0.06)	(0.08)	(0.04)	(0.07)	(0.08)	(0.07)	(0.04)	(0.05)	(0.04)

Significant differences at 5% level between non-scarified seeds (C, for control) and those scarified by passage through rabbit intestines (R) are indicated by asterisk. The effect of scarification was tested on the following irrigation treatments: pH↓sal↓: pH 7, 0.02 g NaCl/L; pH↓sal↑: pH 7, 0.04 g NaCl/L; pH↑sal↓: pH 8, 0.02 g NaCl/L; pH↑sal↑: pH 8, 0.04 g NaCl/L (high moisture: 2 ml/week, low moisture: 1 ml/week); C0: rainwater passed through a dune soil after the removal of *C. edulis*; C1: rainwater passed through a dune soil after the removal of *C. edulis* 1.5 yrs ago; C3: rainwater passed through a dune soil after the removal of *C. edulis* 3 yrs ago; and N: rainwater from a dune soil never affected by C. edulis. Sal = salinity.

**Table 5:** Results of one-way ANOVA testing the effects of scarification on total germination Gt, cumulative germination AS and root growth of *Carpobrotus edulis* in pure cultures.

	C0		C1		C3		N	
	C	R	C	R	C	R	C	R
Gt	22.5*	46.7	22.5*	32.5	10.0*	45.0	17.5	20.0
	(6.2)	(3.3)	(4.7)	(4.8)	(4.0)	(5.0)	(4.7)	(7.0)
As	0.26*	0.64	0.36	0.39	0.20*	0.42	0.40	0.34
	(0.12)	(0.11)	(0.06)	(0.07)	(0.07)	(0.01)	(0.10)	(0.11)
Root	3.1*	3.6	2.9	2.9	3.0	3.0	2.7*	3.5
	(0.1)	(0.2)	(0.4)	(0.3)	(0.1)	(0.2)	(0.1)	(0.4)

Significant differences at 5% level between non-scarified seeds (C, for control) and those scarified by passage through rabbit intestines (R) are indicated by asterisk. The effect of scarification was tested on the following irrigation treatments: C0: rainwater passed through a dune soil after the removal of *C. edulis*; C1: rainwater passed through a dune soil after the removal of *C. edulis* 1.5 yrs ago; C3: rainwater passed through a dune soil after the removal of *C. edulis* 3 yrs ago; and N: rainwater from a dune soil never affected by C. edulis. Sal = salinity.

### Competition

Our results showed no competition between seeds of *C. edulis* and *M. littorea* as indicated by non-significant differences (*P*≤0.05) between pure and mixed seed cultures.

### Allelopathy potential

**Figure 4:**
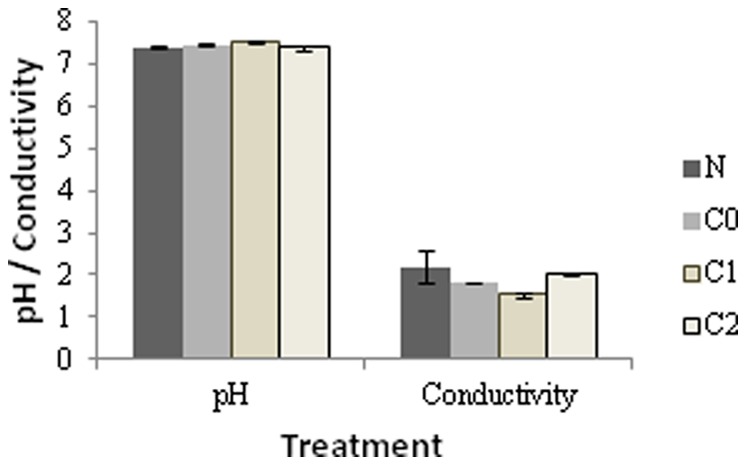
pH and Conductivity levels of rainwater.Rainwater passed through a dune soil after the removal of *C. edulis* at the start of the experiment.

**Figure 5:**
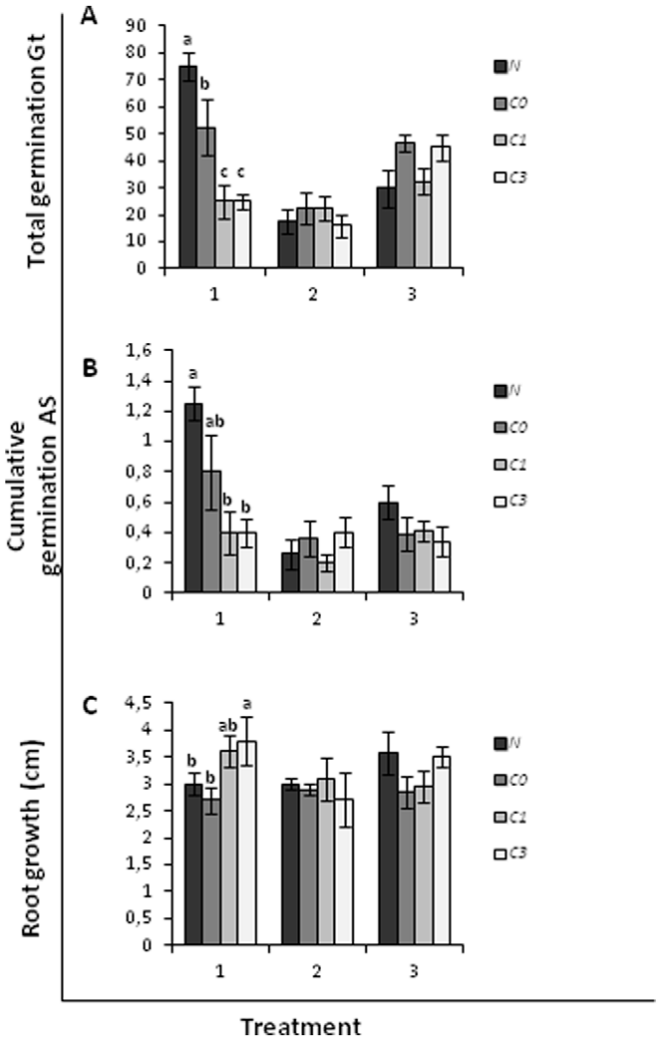
Total germination rate Gt (A), cumulative germination AS (B) and root growth (C) of the native species *Malcolmia littorea* and the invasive plant *Carpobrotus edulis* (scarified and not scarified) as affected by C. edulis litter.

We found no significant differences on pH or conductivity of rainwater solutions (fig. 4).The rainwater passed through the soil surface of sites invaded by *C. edulis* significantly affected the germination and early root growth of *Malcolmia littorea*, reducing its total germination (Gt) and cumulative germination (AS) to 30–67% and 36–68%, respectively (Fig. 5 A, B), of control values and root growth to 6–29% (Fig. 5C). This effect on germination was stronger in areas from which *C. edulis* was removed long ago (1.5 and 3 years) than on invaded areas from which *C. edulis* has just been removed. However, germination and root growth of *Carpobrotus* was never affected by treatments (Fig. 5).

## Discussion

### Effects of moisture, pH and salinity

Annual dune species germinate in autumn or spring, in the rainy seasons when there is more water in the soil, and the salt content decreases [1], [64]. To start germination, enough water of sufficient quality must be available. The water softens the seed coat so the radicle can emerge more easily and also solubilizes nutrients [65]. But a high salt content can block the germination process by the osmotic effect, drawing water from seeds [66]. It is known that when *Carpobrotus* invades coastal habitats, it modifies soil salinity, pH or moisture content [27], [28], [29], [20], [21].

The germination process of *M. littorea* is stimulated by high moisture and low salinity (Novoa and González, unpublished). This corresponds to the negative relationship of the seedling growth of dune species with increasing salinity and decreasing moisture [67], [68], [69]. Our results indicate, as salinity never appeared significant in the models, that moisture is the more important factor determining the seed germination of this native species. *Carpobrotus edulis*, on the other hand, seems to be rather plastic terms of response to soil conditions during germination; in general, high plasticity is typical of many invasive plants [70], [71]. Imperceptible changes in the physicochemical and biological soil conditions therefore put the native species *M. littorea* at disadvantage against *C. edulis*.

The pH level is also one of the most important limiting factors of available soil nutrients [72]. If the two factors are tested separately, both affect the radicle growth of *M. littorea*, which is stimulated by relatively low salinity and pH. Similarly, the shoot growth of *C. edulis* increased at high moisture (Novoa and González unpublished). Testing the salinity, moisture and pH level together, however, suggests that moisture is the major factor determining early seedling growth of both the native and invasive species.

### Scarification

Dispersal of seeds via the digestive tract of herbivores, endozoochory, has long been investigated [73]. Endozoochory may become an efficient mechanism for the spread of non-native species into new environments and of their dispersal in invaded areas [37], [74], [75]. *Carpobrotus edulis* produces a fleshy indehiscent fruit with small seeds during spring, a period of the year when other food is scarce and in habitats in which no native species bear fleshy fruit [37]. Ripe *Carpobrotus edulis* fruit remains on the plant until it is eaten by a variety of mammals, including rabbits, deers… [36], [38].

The role of European rabbit (*Oryctolagus cuniculus*) as seed disperser has been studied from a quantitative perspective mainly in the Mediterranean continental environments [37], [73] and its role in dispersing *C. edulis* seed has been documented [38], [76]. Our results show that rabbits not only disperse *C. edulis* to new locations as reported previously [37], [38], [73], [76] but also favor its invasiveness by increasing the probability that seeds will germinate and establish.

### Allelopathy

Allelopathy is defined as an interference mechanism in which live or dead plant materials, including plant litter during the decomposition process, release biochemical compounds that exert an effect on associated plants [77]. Its action promoted the formulation of the “novel weapons hypothesis” that states that some invaders possess biochemical compounds that function as unusually powerful allelopathic agents, or as mediators of new plant–soil microbial interactions [78]. Moreover, plant litter has been shown to exert effect on germination in various ecosystems [79] that, depending on situation may inhibit [80], [81] or increase seedling recruitment [82].

The natural solutions assayed on the native and invasive seeds showed an inhibition effect of *C. edulis* litter against *M. littorea* but not against *C. edulis*. When *C. edulis* invades coastal habitats, it grows between and on native vegetation, creating a monospecific cloak in just a few years and changing the substrate characteristics [27]. Wardle et al. [83] proposed that in communities where the nature of the soil biochemistry is determined by a dominant plant species, effective and consistent allelopathic inhibition of one species by another is more likely to occur. So the allelopathic inhibition of native plant establishment by *C. edulis* litter was expected.

An interesting result is that the germination of *M. littorea* was more suppressed by *C. edulis* litter accumulated a long time ago than by *C. edulis* litter recently accumulated. This can be most likely explained by the tissues of *C. edulis* decomposing slowly, during which process the substrate is modified [29]. Plants producing tissues with slow litter decomposition contain high levels of secondary metabolites, and could therefore conceivably have a greater allelopathic potential [83]. Thus, the great production of litter by *C. edulis* [29] could ensure the accumulation of allelochemicals in the previously invaded area as the litter is decomposing.

The experimental approaches frequently used for studying allelopathy have drawn considerable criticism from many plant ecologists [77] since (i) it is difficult to correlate the concentration of chemicals used in the extracts with those in nature, (ii) the soil can significantly deactivate secondary metabolites [84] and (iii) other factors such as ion concentration or pH might affect seed germination. We believe the effects observed in *M. littorea* have an allelopathic basis because the concentrations of secondary metabolites are the same as those which would occur in nature, the extracts used in this study are collected directly from rainwater in the soil and we found no differences in pH or conductivity of the solutions. But the effect of biochemicals can vary dramatically among different species [84]. Thus the response of other native species is crucial to understand the allelopathy potential of *C. edulis*.

### Implications for restoration

Our study shows that the invasive species *C. edulis* exhibits features that make it a better colonizer of sand dunes than the coocurring native species *M. littorea*. Allelopathic effects, the ability to establish in drier microsites and efficient endozoochory by rabbits are among the mechanisms allowing *C. edulis* to invade. These facts may, however, provide some insights into difficulties encountered by managers dealing with this species invasion. In the study area, removal projects have been carried out in order to restore invaded dunes and have failed (Novoa & Gonzalez, personal observation). Our results indicate that these removal projects may not be sufficient due to, among other things, the allelopathic effect of the litter that remains on the restored areas after the removal of the invasive plant; its negative effects on germination of the native species *M. littorea* may manifest long after *C. edulis* is removed. In addition, rats and rabbits, the primary seed dispersers of *Carpobrotus* sp, can disperse the seeds up to one kilometre away from the fruiting plant, improving its establishment [31]. This, together with the efficient scarification of *C. edulis* seeds by mammals further constrains eradication efforts.
